# The impact of opioid use on human and health care costs in surgical patients

**DOI:** 10.1016/j.sopen.2019.10.001

**Published:** 2020-01-11

**Authors:** Al-Faraaz Kassam, Young Kim, Alexander R. Cortez, Vikrom K. Dhar, Koffi Wima, Shimul A. Shah

**Affiliations:** Cincinnati Research in Outcomes and Safety in Surgery (CROSS), Department of Surgery, University of Cincinnati College of Medicine, Cincinnati, OH

## Abstract

**Background:**

Preoperative narcotic use impacts hospital cost and outcomes in surgical patients, but the underlying reasons are unclear.

**Methods:**

A single-center retrospective analysis was performed on surgical patients admitted with intestinal obstruction (2010–2014). Patients were grouped into active opioid and nonopioid user cohorts. *Active opioid use* was defined as having an opioid prescription overlapping the date of admission. *Chronic opioid use* was defined by duration of use ≥ 90 days. Admission or intervention due to opioid-related illness was determined through consensus decision of 2 independent, blinded clinicians. Primary end point was the effect of active opioid use on hospital resource utilization.

**Results:**

During the study period, 296 patients were admitted with a primary diagnosis of intestinal obstruction. Active opioid users accounted for 55 (18.6%) of these patients, with a median length of opioid use of 164 days (interquartile range 54–344 days). Average length of use was 164 days, with the majority of active users (*n* = 42, 76.4%) meeting criteria for chronic use. A subgroup analysis of active users demonstrated that opioid-related conditions were responsible for 10 admissions (18.2%) and 2 readmissions (3.6%). Among active users requiring surgical intervention, 3 procedures (21.4%) were due to opioid-related illnesses. Median hospital length of stay was 2 days longer (8 vs 6 days) and hospital costs were greater ($12,241 vs $8489) among active users (*P* < .05 each).

**Conclusion:**

Active opioid users are predisposed to avoidable admissions and interventions for opioid-related illnesses. Efforts to address opioid use in the surgical population may improve patient outcomes and health care spending.

## INTRODUCTION

The overuse of prescription opioid analgesics is a growing epidemic in the United States. The National Institute on Drug Abuse estimates that more than 2 million Americans misuse narcotic medications and approximately 467,000 suffer from heroin addiction [1]. Although opioids are integral to the management of acute postsurgical pain [2], misuse of these medications is associated with considerable morbidity and mortality. The manifold adverse effects of prescription opioids have been well documented, including addiction [3], withdrawal [4], narcotic bowel syndrome [5], and death [6]. As opiate prescription and availability continue to skyrocket in the United States [1], the medical benefits of these drugs must be carefully balanced with their negative impact on personal and public health.

The large-scale economic effects of the opioid epidemic have attracted much attention in recent years [7,8]. The Centers for Disease Control and Prevention estimate that opioids cost the United States $78.5 billion each year [9]. In 2016, the US Department of Health and Human Services allocated more than $6 million toward data collection and analysis alone [10]. Although the public health cost of the opioid crisis has been widely acknowledged, few studies have investigated its impact on a patient-level basis. A recent study by Cron et al found that preoperative opioid use was independently associated with increased health care resource utilization in the surgical population [11]. Preoperative opioid use was predictive of prolonged hospital stay, increased readmission rates, and more complications compared with nonusers [11]. Although hospital outcomes associated with increased health care spending have been studied in the surgical population, the underlying cause of these outcomes is unknown.

In the current study, we used small bowel obstruction (SBO) as our model for analyzing how opioids affect outcomes among surgical patients. Two reasons governed this decision. First, the clinical presentation of SBO may be exacerbated or even confounded by concurrent opioid use. Opioids disturb gastrointestinal physiology in various ways, resulting in symptoms including abdominal pain, gastroparesis, bloating, constipation, delayed gastrointestinal transit, and emesis [12]. Both SBO and opioid-induced bowel dysfunction share many common symptoms, creating the possibility for misdiagnosis and medical mismanagement [13]. Second, SBO accounts for a significant percentage of all surgical admissions, with more than 300,000 of these admissions requiring operative intervention annually [14]. As a condition that should be primarily managed by a surgical team [[15], [16], [17]], SBO represents an area where surgeons can make a profound difference in health care expenditure.

We hypothesized that opioid use confounds clinical decision making and increases health care resource utilization in the surgical population. Primary end points were total direct cost and hospital length of stay (LOS). A subgroup analysis of active opioid users was performed to determine how opioid use affected clinical presentation among these patients to understand the precise reasons for this finding.

## METHODS

A retrospective cohort study was performed for patients admitted with a primary diagnosis of intestinal obstruction between January 1, 2010, and December 31, 2014. Patients were identified via *International Classification of Diseases*, *version 9* (*ICD-9*) codes 560.0–560.9 through the University HealthSystem Consortium (UHC) Clinical Database/Resource Manager (CDB/RM). The UHC CDB/RM is a data set collected by 118 academic medical centers and nearly 300 of their associated hospitals. This data set includes information on patient demographics, financial information, procedural data, and *ICD-9* diagnoses. The following patient characteristics were obtained: age, sex, race, and severity of illness (SOI) scores. SOI scores were calculated using an ordinal scale with 4 levels for severity. Characteristics included stage of principal diagnosis, complications of the principal condition, concurrent interacting conditions that affect the hospital course, dependency on hospital staff, extent life support procedures, rate of recovery, and impairment remaining after therapy. After *ICD-9* code search through the UHC CDB/RM, patient identifiers were used to perform a chart review through our institutional electronic medical records (EPIC, Madison, WI) at the University of Cincinnati Medical Center. This second source was used to collect data on past and current opioid use, and details regarding each admission and surgical intervention.

Patients were divided into opioid-naive and active opioid user cohorts. *Active opioid use* was defined as having an active prescription interval for narcotic medications overlapping the date of admission. For active opioid users, the patient-specific characteristics collected included the opioid type, quantity dispensed, frequency of use, length of use, and whether multiple opioids were being used concurrently. *Chronic opioid use* was defined as length of opioid use exceeding 90 days or longer, as previously described [18]. Within the active user cohort, admission or intervention due to opioid-related illness was determined through consensus decision of 2 blinded independent clinicians. Briefly, data collection was performed by 2 members of the team, including clinical course and operative reports. These documents were then given to 2 separate, blinded clinicians to minimize the potential for subjective bias [[19], [20], [21], [22], [23]]. Opioid-related admission or intervention was determined through consensus decision.

Our data set was organized according to date of admission or readmission. The following hospital-specific characteristics were obtained: total LOS, total direct cost, total cost, 30-day readmission dates, and overall mortality. Costs prior to admission were removed from our data analysis, and total direct costs (defined as admission to discharge) were generated based on encounter-specific data [24,25]. Total cost was calculated as the sum of total direct cost plus any costs of readmission.

Data are described as median values, interquartile range (IQR) for continuous variables, and percentages for categorical variables. For categorical variables, nominal variables were analyzed using Pearson *χ*^2^ test, and ordinal variables were analyzed via Mantel–Haenszel *χ*^2^ test. Univariate analysis was performed using the nonparametric Wilcoxon rank-sum test. Multivariate analysis was performed via gamma regression techniques to determine predictors of total cost and by Poisson regression analyses for total LOS. Models were adjusted for the following covariates: age, sex, race, SOI scores, insurance type, source of admission, active opioid use, and length of use. Statistical significance was set at *P* value less than .05. All analyses were performed using statistical packages SAS 9.4 and JMP Pro 11 (SAS Institute, Cary, NC). This study was approved by the University of Cincinnati Institutional Review Board.

## RESULTS

### Patient Characteristics

A total of 271 patients were admitted to our institution with a primary diagnosis of intestinal obstruction during the study period (Table 1). The active opioid user cohort comprised 18.6% (*n* = 55) of all patient encounters. Active users and opioid-naive patients were not significantly different in age, sex, race, or severity of illness.

**Table 1:** Characteristics of patients admitted with intestinal obstruction from 2010 to 2014

	Active opioid users		Non-opioid users		
Characteristic	*N*/median	%/IQR	*N*/median	%/IQR	*P* value
Patients	47	(17.3%)	224	(82.7%)	
Encounters	55	(18.6%)	241	(81.4%)	
Age (y)	54	(46–67)	57	(46–69)	NS
Sex					NS
Male	21	(38.2%)	116	(48.1%)	
Female	34	(61.8%)	125	(51.9%)	
Race					NS
White	35	(63.6%)	124	(51.5%)	
Black	18	(32.7%)	105	(43.6%)	
Hispanic	0	(0.0%)	0	(0.0%)	
Asian	0	(0.0%)	2	(0.8%)	
Other	2	(3.6%)	10	(4.1%)	
Severity of illness					NS
Minor	4	(7.3%)	35	(14.5%)	
Moderate	15	(27.3%)	78	(32.4%)	
Major	25	(45.5%)	71	(29.5%)	
Extreme	11	(20.0%)	57	(23.7%)	

### Hospital Outcomes and Resource Utilization

Hospital outcomes and cost-related variables are detailed in Table 2. On univariate analysis, active opioid users demonstrated a prolonged hospital LOS (8 vs 6 days) and greater total cost ($12,241 vs $8489) compared to nonusers (*P* < .05 each). Thirty-day readmission rates, mortality rates, and total direct cost were not significantly different between the 2 cohorts. A multivariate analysis was performed to identify predictors of total cost and LOS (Table 3). Only SOI scores were predictive of total cost and LOS (*P* < .001 each). Active opioid use did not persist as a predictor of either outcome on multivariate analysis, likely as a result of the relatively small number of patients in each cohort over the study period.

**Table 2:** Hospital outcomes of patients admitted with intestinal obstruction from 2010 to 2014

	Active opioid users		Non-opioid users		
Hospital outcome	*N*/median	%/IQR	*N*/median	%/IQR	*P* value
LOS (d)	8	(5–14)	6	(4–11)	.04
Total direct cost ($)	$9948	($4296–$23,056)	$8003	($3731–$16,047)	NS
Total cost ($)	$12,241	($4995–$30,817)	$8489	($4111–$17,437)	.04
30-d readmission	14	(25.5%)	41	(17.0%)	NS
Mortality	0	(0.0%)	9	(3.7%)	NS

**Table 3:** Predictors of total cost and hospital LOS on multivariate analysis

	Predictors of total cost			Predictors of LOS		
Characteristic	Relative risk	95% CI	*P* value	Relative risk	95% CI	*P* value
Age (y)	1.00	(0.99–1.01)	NS	1.00	(0.99–1.00)	NS
Race			NS			NS
White	Ref.			Ref.		
Black	0.99	(0.75–1.23)		1.10	(0.89–1.32)	
Asian	0.39	(0.01–0.94)		0.42	(0.01–1.29)	
Other	0.98	(0.40–1.57)		1.16	(0.61–1.71)	
Sex			NS			NS
Male	Ref.			Ref.		
Female	1.02	(0.78–1.27)		0.97	(0.79–1.16)	
Severity of illness			< .01			< .01
Minor	Ref.			Ref.		
Moderate	1.43	(0.88–1.98)		1.33	(0.75–1.91)	
Major	2.74	(1.70–3.78)		2.04	(1.19–2.89)	
Extreme	8.28	(4.89–11.68)		4.41	(2.58–6.24)	
Insurance type			NS			NS
Private	Ref.			Ref.		
Government	0.77	(0.55–1.00)		0.96	(0.73–1.18)	
Other	1.17	(0.52–1.83)		1.17	(0.62–1.71)	
Admission source			NS			NS
Home	Ref.			Ref.		
ER	0.80	(0.50–1.10)		0.86	(0.57–1.15)	
Hospital	0.91	(0.63–1.20)		1.01	(0.76–1.25)	
Other	0.95	(0.33–1.56)		0.77	(0.37–1.16)	
Active opioid use	0.82	(0.15–1.50)	NS	0.80	(0.32–1.28)	NS

### Subgroup Analysis of Active Opioid Users

Table 4 details characteristics of the active opioid user cohort. Among active users, the most common narcotic taken was oxycodone (*n* = 43, 66.2%), followed by hydrocodone (*n* = 6, 9.2%) and tramadol (*n* = 5, 7.7%). Active users were dispensed a median 60 tablets per prescription (IQR 30–157 tablets). The median length of use was 164 days (IQR 54–344 days) with a frequency of use of 6 times daily (IQR 4–6 tablets per day). The majority of active users met criteria for chronic opioid use (*n* = 42, 76.4%). A significant percentage of patients (*n* = 10, 18.2%) were prescribed multiple narcotics at time of admission. After chart review by 2 blinded independent clinicians, 10 admissions (18.2%) and 2 readmissions (3.6%) were ascribed to opioid-related illnesses, and no small bowel or colonic pathology was noted. Of the remaining 45 encounters, 12 (21.8%) were due to a colonic obstruction, whereas 33 (60%) were due to a small bowel obstruction. Etiology of obstruction was attributed to adhesive disease in 32 (58.2%) encounters, hernia in 3 (5.5%), cancer in 6 (10.9%), and stricture in 4 (7.3%). Among active opioid users requiring inpatient admission, 14 required surgical intervention (25.5%). Opioid-related illnesses were responsible for 3 of these 14 interventions (5.5%) according to consensus decision, which was defined as current opioid use requiring an admission without imaging findings of a cause of obstruction or negative operative exploration. All 3 operations were exploratory laparotomy with a diagnosis of partial small bowel obstruction, with inability to identify adhesive disease during the operation.

**Table 4:** Subgroup analysis of active opioid users (*n* = 47).

Characteristic	*N*/median	%/IQR
Opioid type		
Buprenorphine	0	(0.0%)
Fentanyl	2	(3.1%)
Hydrocodone	6	(9.2%)
Hydromorphone	4	(6.2%)
Methadone	1	(1.5%)
Morphine	4	(6.2%)
Oxycodone	43	(66.2%)
Tramadol	5	(7.7%)
PRN	56	(86.2%)
Dispense quantity (tablet)	60	(30–157)
Frequency of use (tablet/d)	6	(4–6)
Length of use (d)	164	(54–344)
Multiple opioid use	10	(18.2%)
Chronic opioid use	42	(76.4%)
Opioid-related admissions	10	(18.2%)
Opioid-related readmissions	2	(3.6%)
Surgical intervention	14	(25.5%)
Opioid-related intervention	3	(5.5%)

## DISCUSSION

As the opioid epidemic continues to plague our health care systems, the annual federal spending on opioid-related disorders has skyrocketed [9,10]. These costs encompass loss of workforce productivity, criminal justice–related matters, and health care expenditure, with the majority of the burden attributed to health care spending [9]. Although the adverse effects of opioid analgesics have been well described, recent studies have shown that opioid use prior to surgery is also predictive of increased health care resource utilization postoperatively [11]. Our results validate this finding, with a significant difference in total cost between active and nonopioid users, and suggest an explanation for this finding—active opioid users may be predisposed to unnecessary hospital admission, readmission, and surgical intervention.

The opioid crisis remains widely prevalent at our tertiary care institution. One in every 5 patients admitted with SBO during the study period was an active opioid user. These patients were prescribed an average of 60 tablets with significant variance in the number of tablets allotted (IQR 54–344). Furthermore, the average length of use was 164 days, with most active users meeting criteria for chronic opioid use. Among active users, 18.2% were taking multiple narcotics at time of admission. Although these statistics may seem staggering, they are consistent with studies in similar patient populations [11,26].

Among opioid users admitted with a presumptive diagnosis of SBO, a significant number were found to have an alternative explanation for their condition. We found that most misdiagnoses were attributable to opioid-induced bowel dysfunction, with presenting symptoms of abdominal pain, nausea, and emesis. Although many of these patients were discharged after their clinical pictures were clarified, 3 underwent exploratory laparotomy. Two were presumed to have adhesive disease due to the severity of their symptoms, and 1 patient carried a preoperative diagnosis of small bowel intussusception. Operative exploration failed to find any culpable lesion in any of the 3 patients. Not only were these patients subject to an avoidable laparotomy, they were also exposed to risks of iatrogenic injury; nosocomial infection; considerable health care expenditure; and, ironically, postsurgical pain. Further work is still needed to differentiate opioid induced bowel dysfunction to prevent unnecessary surgery preoperatively. Given that opioid-induced bowel dysfunction is a nonoperative diagnosis, by addressing narcotic use in the surgical population, surgeons can improve patient outcomes and overall health care spending.

These data highlight 3 areas where quality control of narcotic prescription policy may improve outcomes. First, there must be efforts to reduce variability in opioid prescription. Other studies have also noted wide variations in opioid dosage across common general surgery procedures [27]. By imposing regulations on the number of narcotics prescribed on a per procedure basis, patients may be at less risk for becoming chronic users. Second, patients should be informed that prolonged opioid use may increase their risk of avoidable admissions and surgical interventions. Third, discharging physicians should be cognizant of the patient's current medications prior to prescribing any additional narcotics. Although opioid polypharmacy may be appropriate in certain situations (eg, chronic pain), this approach results in significant exposure to medication-related harm [28].

There are several limitations to the present study. One limitation is its retrospective nature. As with all retrospective analyses, these data are subject to measuring error and the potential for selection bias. Second, our tertiary care medical center is a teaching institution. Patient admissions and surgical interventions are subject to approval by attending-level surgeons, but resident and fellow trainees are present at all levels of care. Third, active opioid use and chronic opioid use were defined through prescriptions within our electronic medical record system. Although these charts are reliable in capturing all active prescriptions at our institution, patient polling may be more accurate in gathering data related to opioid use patterns. Fourth, our results are derived from a single institution. This allows for significant granularity in patient admission and operative intervention, but further studies are necessary before extrapolating these results to other medical centers.

## CONCLUSION

Opioid analgesics are an integral part in managing postoperative pain, but their misuse and overuse have severe personal and public health implications. Among the surgical population, active opioid users may be subject to admissions and surgical intervention for opioid-related illnesses. By addressing the potential harms of opioid use in the preoperative phase and standardizing narcotic prescriptions postoperatively, prescribing physicians may improve patient outcomes and overall health care spending.

## Author Contribution

AK, YK, ARC, and VKD contributed significantly with study design, data collection, data analysis, interpretation of results, drafting of manuscript, and revision of the manuscript; KW contributed to statistical analysis, and SAS contributed to study design, interpretation of results, and drafting and revision of the manuscript. All authors approved the final version of the paper.

## Conflict of Interest

We wish to confirm that there are no known conflicts of interest associated with this publication and there has been no significant financial support for this work that could have influenced its outcome.

## Funding Sources

This research did not receive any funding.

## References

[1] Centers for Disease Control and Prevention (2016). Wide-ranging online data for epidemiologic research (WONDER). http://wonder.cdc.gov.

[2] Oderda G. (2012). Challenges in the management of acute postsurgical pain. Pharmacotherapy.

[3] Ling W., Mooney L., Hillhouse M. (2011). Prescription opioid abuse, pain and addiction: clinical issues and implications. Drug Alcohol Rev.

[4] Farrell M. (1994). Opiate withdrawal. Addiction.

[5] Grunkemeier D.M., Cassara J.E., Dalton C.B., Drossman D.A. (2007). The narcotic bowel syndrome: clinical features, pathophysiology, and management. Clin Gastroenterol Hepatol.

[6] MED Calculator (2017). Ohio Automated Rx Reporting System [Internet]. https://www.ohiopmp.gov/Portal/MED_Calculator.aspx.

[7] Burke D.S. (2016). Forecasting the opioid epidemic. Science.

[8] Florence C.S., Zhou C., Luo F., Xu L. (2016). The economic burden of prescription opioid overdose, abuse, and dependence in the United States, 2013. Med Care.

[9] (2016). Opioid epidemic costs U.S. $78.5 billion annually: CDC. Washington, DC, U.S. News and world report. http://health.usnews.com/health-care/articles/2016-09-21/opioid-epidemic-costs-us-785-billion-annually-cdc.

[10] Chang H.Y., Daubresse M., Kruszewski S.P., Alexander G.C. (2014). Prevalence and treatment of pain in EDs in the United States, 2000 to 2010. Am J Emerg Med.

[11] Cron D.C., Englesbe M.J., Bolton C.J., Joseph M.T., Carrier K.L., Moser S.E. (2016). Preoperative opioid use is independently associated with increased costs and worse outcomes after major abdominal surgery. Ann Surg.

[12] Panchal S.J., Muller-Schwefe P., Wurzelmann J.I. (2007). Opioid-induced bowel dysfunction: prevalence, pathophysiology and burden. Int J Clin Pract.

[13] Drossman D., Szigethy E. (2014). The narcotic bowel syndrome: a recent update. Am J Gastroenterol Suppl.

[14] Maung A.A., Johnson D.C., Piper G.L., Barbosa R.R., Rowell S.E., Bokhari F. (2012). Evaluation and management of small-bowel obstruction: an Eastern Association for the Surgery of Trauma practice management guideline. J Trauma Acute Care Surg.

[15] Aquina C.T., Becerra A.Z., Probst C.P., Xu Z., Hensley B.J., Iannuzzi J.C. (2016). Patients with adhesive small bowel obstruction should be primarily managed by a surgical team. Ann Surg.

[16] Malangoni M.A., Times M.L., Kozik D., Merlino J.I. (2001). Admitting service influences the outcomes of patients with small bowel obstruction. Surgery.

[17] Schwab D.P., Blackhurst D.W., Sticca R.P. (2001). Operative acute small bowel obstruction: admitting service impacts outcome. Am Surg.

[18] Scherrer J.F., Svrakic D.M., Freedland K.E., Chrusciel T., Balasubramanian S., Bucholz K.K. (2014). Prescription opioid analgesics increase the risk of depression. J Gen Intern Med.

[19] Hrobjartsson A., Thomsen A.S., Emanuelsson F., Tendal B., Hilden J., Boutron I. (2012). Observer bias in randomised clinical trials with binary outcomes: systematic review of trials with both blinded and non-blinded outcome assessors. BMJ.

[20] Hrobjartsson A., Thomsen A.S., Emanuelsson F., Tendal B., Hilden J., Boutron I. (2013). Observer bias in randomized clinical trials with measurement scale outcomes: a systematic review of trials with both blinded and nonblinded assessors. CMAJ.

[21] Kahan B.C., Cro S., Dore C.J., Bratton D.J., Rehal S., Maskell N.A. (2014). Reducing bias in open-label trials where blinded outcome assessment is not feasible: strategies from two randomised trials. Trials.

[22] Poolman R.W., Struijs P.A., Krips R., Sierevelt I.N., Marti R.K., Farrokhyar F. (2007). Reporting of outcomes in orthopaedic randomized trials: does blinding of outcome assessors matter?. J Bone Joint Surg Am.

[23] Savovic J., Jones H.E., Altman D.G., Harris R.J., Juni P., Pildal J. (2012). Influence of reported study design characteristics on intervention effect estimates from randomized, controlled trials. Ann Intern Med.

[24] Quillin R.C., Wilson G.C., Wima K., Hohmann S.F., Sutton J.M., Shaw J.J. (2014). Neighborhood level effects of socioeconomic status on liver transplant selection and recipient survival. Clin Gastroenterol Hepatol.

[25] Wilson G.C., Quillin R.C., Wima K., Sutton J.M., Hoehn R.S., Hanseman D.J. (2014). Is liver transplantation safe and effective in elderly (>/=70 years) recipients? A case-controlled analysis. HPB (Oxford).

[26] Jiang X., Orton M., Feng R., Hossain E., Malhotra N.R., Zager E.L. (2016). Chronic opioid usage in surgical patients in a large academic center. Ann Surg.

[27] Hill M.V., McMahon M.L., Stucke R.S., Barth R.J. (2016). Wide Variation and Excessive dosage of opioid prescriptions for common general surgical procedures. Ann Surg.

[28] Giummarra M.J., Gibson S.J., Allen A.R., Pichler A.S., Arnold C.A. (2015). Polypharmacy and chronic pain: harm exposure is not all about the opioids. Pain Med.

